# Do yoga and meditation moderate the relationship between negative life events and depressive symptoms? Analysis of a national cross-sectional survey of Australian women

**DOI:** 10.3389/fpsyg.2023.1218976

**Published:** 2023-09-05

**Authors:** Romy Lauche, Dennis Anheyer, Lisa A. Uebelacker, David Sibbritt, Jon Adams, Holger Cramer

**Affiliations:** ^1^National Centre for Naturopathic Medicine, Southern Cross University, Lismore, NSW, Australia; ^2^Institute for General Practice and Interprofessional Care, University Hospital Tübingen, Tübingen, Germany; ^3^Robert Bosch Center for Integrative Medicine and Health, Bosch Health Campus, Stuttgart, Germany; ^4^Department for Psychology and Psychotherapy, Witten/Herdecke University, Witten, Germany; ^5^Department of Psychiatry and Human Behaviour, Alpert Medical School of Brown University, Providence, RI, United States; ^6^Butler Hospital, Providence, RI, United States; ^7^School of Public Health, Faculty of Health, University of Technology Sydney, Sydney, NSW, Australia

**Keywords:** depression, stress, negative life events, coping, yoga, meditation

## Abstract

**Objectives:**

This study aims to examine the role of yoga/meditation in the relationship between negative life events, stress and depression.

**Methods:**

The Australian Longitudinal Study on Women’s Health (ALSWH) surveyed 7,186 women aged 36–43 years (mean age 39.2 years; 57.2% university degree) in 2015. Mediation and moderation analyses were conducted to examine whether yoga/meditation practice moderated those relationships.

**Results:**

Yoga/meditation was practiced by 27.5% of participants, 33.2% reported negative life events in the past 12 months, and 24% had clinical depression. Perceived stress partially mediated the association between negative life events and depressive symptoms (B = 6.28; 95%CI 5.65; 6.92). Social support (B = −0.38; 95%CI −0.54; −0.23) and optimism (B = −0.25;95%CI −0.31; −0.18) moderated the association between stress and depressive symptoms. Yoga/meditation practice moderated the direct association between negative life events and depressive symptoms (B = −0.92; 95%CI −1.67; −0.18).

**Conclusion:**

Yoga/meditation use was a significant moderator of the relationship between negative life events and depression. Yoga/mediation use did not act via reducing perceived stress, but instead was found to dampen the influence of negative life events on depression directly. More research on how yoga has an impact on depression is warranted.

## 1. Introduction

Yoga is a complex practice that is comprised of advice for an ethical lifestyle, physical activity, and breathing and meditation exercises (Feuerstein, 1998; De Michelis, 2008). Although yoga originally evolved as a spiritual practice, in the 20th and 21st century, it has become a popular means of promoting physical and mental health and wellbeing (Feuerstein, 1998; De Michelis, 2008), especially outside of India.

An estimated 21 million American adults report that they have practiced yoga in the last 12 months in 2012, and 9.8 million have used some form of meditation at the same time (Cramer et al., 2016a). Prevalence rates in other (western) countries are similar (Xue et al., 2007; Cramer, 2015). A large number of people practice yoga or meditation specifically for their mental health and well-being (e.g., to reduce depression, anxiety and stress; Cramer et al., 2016b). Clinical evidence suggests that the use of yoga might be beneficial for a variety of mental health conditions, including depression. Systematic reviews have revealed preliminary evidence for efficacy and safety of yoga for major depressive disorders (Cramer et al., 2017), as well as for individuals with depressive symptoms but without a formal diagnosis of a depressive disorder (Cramer et al., 2013a). Further reviews have shown that yoga can be effective and safe for individuals with post-traumatic stress disorder (Cramer et al., 2018).

Yoga commonly includes physical activity and mental exercises, such as relaxation and meditation, although some yoga schools exclusively rely on the physical activity part (Feuerstein, 1998; De Michelis, 2008). Physical activity (Irandoust et al., 2019; Heissel et al., 2023), relaxation (Jia et al., 2020) and also meditation in itself (Jain et al., 2015) can be beneficial for depression.

Negative life events (e.g., the death of family members, physical assault) have been linked to depressive episodes in previous research (Kendler et al., 1999; Hammen, 2005; Risch et al., 2009). Previous research has also indicated that the response to negative life events may be influenced by the perception of stress (Kuiper et al., 1986). Several factors have been identified to protect against depression after stressful events, including social support, and an optimistic view on life (Scheier and Carver, 1992; Auerbach et al., 2011; Marroquin, 2011; Souri and Hasanirad, 2011).

Few studies have examined how or why yoga or meditation might improve depression symptoms. For example, yoga practice may lead to improved regulation of stress reactivity via the hypothalamic–pituitary–adrenal (HPA) axis, and the noradrenergic, serotonergic, and dopaminergic systems, or, on another level, promotion of more adaptive ways of thinking, including increasing acceptance and decreasing self-criticism (Uebelacker et al., 2010). Qualitative studies on yoga support those above theories, as individuals practicing yoga describe that yoga increases their acceptance of life’s burden (Cramer et al., 2013a), and decreases rumination (Kinser et al., 2013a,b). Thus, yoga or meditation may be able to “buffer” the effect of stressful life events on depression in that the yoga practitioner may experience less physiological reactivity, and may be able to be more accepting of changes that occur as a result of the event.

### 1.1. Aims of the study

This study examined the association between negative life events, perceived stress and depressive symptoms; and the role of yoga/meditation practice (as well as potential other factors such as social support, optimism, and physical activity) as potential moderators in the relationship between negative life events and depressive symptoms in young Australian women.

### 1.2. Hypotheses

The following null hypotheses were tested: (1) having experienced a negative life event in the past year is not associated with higher levels of current depressive symptoms. (2) Perceived stress does not mediate the relationship between negative life events and depressive symptoms. (3) Yoga/meditation practice, social support, optimism, and physical activity do not moderate the relationships between negative life events, stress and depression.

The alternative hypotheses thus were: (1) having experienced a negative life event in the past year is associated with higher levels of current depressive symptoms. (2) Perceived stress mediates the relationship between negative life events and depressive symptoms. (3) Yoga/meditation practice, social support, optimism, and physical activity moderate the relationships between negative life events, stress and depression.

## 2. Methods

The analyses reported here were conducted using data from the Australian Longitudinal Study on Women’s Health (ALSWH), which had been designed to assess health and wellbeing and associated factors in Australian women. Women in three different age groups (18–23, 45–50, and 70–75 years) were randomly selected from the national Medicare database in 1996 (Brown et al., 1998), with respondents shown to be broadly representative of the national population of women in the respective age cohorts (Brown et al., 1999). Following the baseline survey, women were assessed via follow-up survey every 3 years. For the sub-study reported here, only data from the 2015 wave were utilized, and analyses focused on the 7,186 women from the cohort born in 1973–1978, these women were between 36 and 43 years of age at the time of the 2015 survey. This cohort was selected because our analyses have shown that the women in this cohort had the highest rate of yoga utilization (data not shown). This cohort consisted of 14,762 women at the start of the longitudinal study. The 7,186 women still participating at the time of the analyzed survey (19 years after the start of the study) thus represent 48.7% of the original cohort (51.3% attrition). The previous surveys were not included in this cross-sectional analysis, nor were data from the other age groups.

### 2.1. Dependent variable

Depressive symptoms were measured using the Center for Epidemiologic Studies Depression Scale (CESD-10; Radloff, 1977). Total scores range from 0 to 30 points, and higher scores indicate higher levels of depression.

### 2.2. Independent variables

Negative life events were measured by asking women whether one of the following events had happened in the past 12 months: death of partner, parent or child; being pushed, grabbed, shoved, kicked or hit; being forced to take part in unwanted sexual activity; or being bullied. For these analyses, we generated a binary variable reflecting whether a participant had experienced at least one major life event in the last year or having experienced none.

Stress was measured using the Perceived Stress Questionnaire for Young Women (PSQ; Bell and Lee, 2003). Participants were asked to rate how stressed they have been in the past 12 months as a result of potential stressors, including health, work, living arrangement, money, and relationships. The total scores range from 0 to 4, with higher scores indicating higher perceived stress.

Social support was measured using the abbreviated Medical Outcomes Study Social Support Index (MOS-SSS-6; Holden et al., 2014) with 6 items on a 5-point Likert-scale. Total average scores ranged from 1 to 5, with higher scores indicating more social support.

The Life Orientation Test (LOT-R; Scheier and Carver, 1985; Scheier et al., 1994) was used to measure optimistic and pessimistic attitudes towards life. For the purposes of this analysis, we used only the optimism scale; scores range from 0 to 12, with higher scores indicating more optimism.

Participating women were asked how often they had used “yoga/meditation” in the last 12 months. Response options included: “never,” “rarely,” “sometimes,” and “often.” In this question, yoga and meditation use were not differentiated; “never” meant that neither yoga nor meditation had been used in the past 12 months; “rarely,” “sometimes,” or “often” meant that either yoga or meditation or both had been used in the past 12 months. For the purposes of these analyses, the categories “never” and “rarely” were combined into one, which served as the “no yoga/meditation use” reference category and compared to “some yoga/meditation use” (when “sometimes” was chosen) and “frequent yoga/meditation use” (when “often” was chosen).

To assess physical activity, women were asked about the time spend on walking and engaging in moderate and vigorous activities during the previous week. Categories of activity were accompanied by descriptors and examples, and time had to be entered in hours and minutes of total activity per week. All entries were recoded into their metabolic equivalents (MET) based on a defined syntax (Brown et al., 2008, 2013) with one MET being defined as energy expenditure at rest, usually equivalent to 3.5 ml of oxygen uptake per kg per minute. A sum score was calculated that represented an estimate of the weekly exercise level. For this sum score, 0 to 30 METs were categorized as sedentary, greater than 30 and less than 600 METs as low physical activity, greater than 600 and less than 1,200 METs as moderate physical activity, and 1,200 or more METs per week as high physical activity. Moderate or higher levels of physical activity were considered as sufficient based on recommendations from WHO.

### 2.3. Statistical analyses

Mediator and moderator analyses were conducted to examine the influence of independent variables on the relationship between negative life events and depressive symptoms. Mediation and moderation analysis were performed using the PROCESS macro (Version 3.0) for SPSS (IBM SPSS Statistics for Windows, release 24.0. Armonk, NY: IBM Corp.), following the procedure recommended by Hayes (2009).

For the analysis of mediating effects, total, direct and indirect effects were estimated using a bootstrap sampling procedure with 10,000 resamples. As discussed by Hayes (2009), the bootstrapping procedure overcomes several limitations (Baron and Kenny, 1986), yielding results that are more accurate and less affected by sample size (Preacher and Hayes, 2004, 2008; Hayes, 2009). To test for moderation, the direct effect of the predictor on the dependent variable, the direct effect of the moderator on the dependent variable, and the interaction effect (predictor x moderator) were entered into the model. The hypotheses mentioned in section 1.2 were tested. Unstandardized point estimates and bias corrected 95% confidence intervals (CI) were calculated (Hayes, 2017).

## 3. Results

### 3.1. Sample

The sample (Table 1) consisted of 7,186 women with a mean age of 39.2 years (range 36–43). The majority of these women were in a relationship (79.7%), and they reported high levels of formal qualifications, with 57.2% reporting a university degree or above. Two thirds (58.8%) of these women lived in major cities, and another 26.6% in inner regional areas. Yoga/meditation was practiced in the last 12 months by one in four women, and they reported occasional use (16.6%), or frequent use (10.9%). Overall half of the women reported sufficient physical activity levels, 31.5% of all women even high activity levels.

**Table 1:** Sociodemographic, and health characteristics of 7,186 Australian women born between 1973 and 1978 participating in the 2015 survey wave.

Variable	Frequency (%) *N* = 7,186	Mean (SD) *N* = 7,186
Age at survey, in years		39.2 (1.5)
Marital status		
Never married	814 (11.9)	
Married/*de facto*	5,433 (79.7)	
Separated/divorced/widowed	571 (8.4)	
Formal qualifications*		
School only	1,055 (15.6)	
Trade/apprentice/diploma	1856 (27.3)	
University/Higher Degree	3,889 (57.2)	
Area		
Major cities	3,974 (58.8)	
Inner Regional	1801 (26.6)	
Outer regional	829 (12.3)	
Remote	157 (2.3)	
Yoga/meditation practice		
Never/rarely	5,179 (72.5)	
Sometimes	1,183 (16.6)	
Often	780 (10.9)	
Physical activity level		
Sedentary	902 (14.3)	
Low	2075 (32.9)	
Moderate	1,346 (21.3)	
High	1990 (31.5)	
Negative life events, last 12 months	2,343 (33.2)	
Depressive symptoms, CESD-10		6.6 (5.5)

* 51 women (0.8%) reported that they have not finished high school, they were included in the school only category which served as the reference category.

One third of women (33.2%) reported that they had experienced at least one negative life event in the past 12 months. The average depression score was 6.6 ± 5.5 on the CESD-10 scale, and based on their responses one in four women (24%) were classified as having clinical depression levels (defined as CESD-10 ≥ 11).

**Figure 1:**
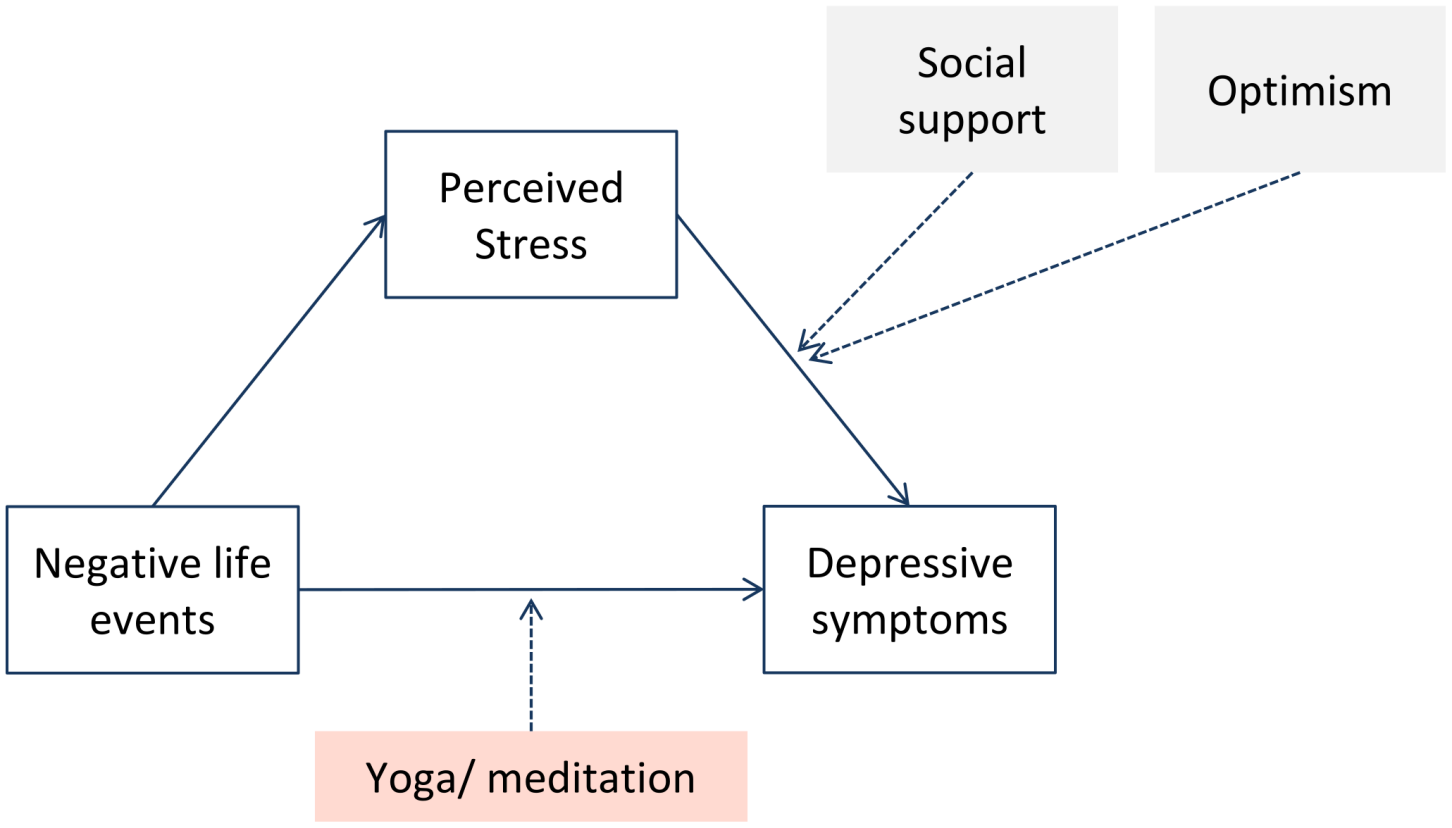
Moderating effects of yoga/meditation on the relationship between life events and depression. The direct path between negative live events and depressive symptoms as well as mediation via stress are shown using solid lines, moderation using dashed lines.

Table 2 shows the correlations between all study variables, and it shows that all variables except for physical activity are correlated with negative life events. Correlation coefficients however are rather small, with a maximum of Pearson’s r = 0.201. Figure 1 shows the final model of mediation and moderation of the relationship between negative life effects and depression by the variables stress, social support, optimism and yoga/meditation use. As can be seen from Figure 1, yoga moderates the direct path between life events and depression, rather than moderating the effects via stress. The contribution of each variable on the relationship between negative life events and depressive symptoms is presented in Table 3, and described in the following sections.

**Table 2:** Correlations between study variables.

	Negative life events	Depression	Perceived stress	Social support	Optimism	Yoga use	Physical activity
Negative life events							
Depression	0.191**						
Perceived stress	0.201**	0.513**					
Social support	−0.141**	−0.421**	−0.306**				
Optimism	−0.122**	−0.526**	−0.292**	0.303**			
Yoga use	0.036**	0.009 ^ns^	0.062**	0.018 ^ns^	0.106**		
Physical activity	0.009 ^ns^	−0.166**	−0.109**	0.074**	0.121**	0.165**	

**: significant; ns: not significant.

**Table 3:** Moderation of the relationship between negative life events, stress, and depression (Path 1), and between negative life events, and depression (Path 2) through optimism, social support, and yoga/meditation use.

Predictor	B	SE	Bootstrapping confidence interval (BC*)		*p*-value
			Lower 2.5%	Higher 2.5%	
Path 1: Negative life events → stress → depressive symptoms					
Outcome stress [R^2^ = 0.157, *F*(5;6,692) = 248.782]					
Negative life events (indirect effect)	0.343	0.072	0.202	0.484	<0.001
Social Support	−0.143	0.010	−0.163	−0.122	<0.001
Negative life events × Social Support	−0.022	0.016	−0.054	0.010	0.182
Optimism	−0.054	0.004	−0.062	−0.046	<0.001
Negative life events × Optimism	−0.008	0.007	−0.021	0.005	0.215
Outcome depressive symptoms [R^2^ = 0.467, *F*(6;6,691) = 977.812]					
Negative life events (direct effect)	0.616	0.106	0.408	0.825	<0.001
Stress	6.284	0.325	5.646	6.921	<0.001
Social Support	−0.766	0.942	0.952	−0.582	<0.001
Stress × Social Support	−0.382	0.079	−0.537	−0.227	<0.001
Optimism	0.611	0.038	−0.686	−0.536	<0.001
Stress × Optimism	−0.250	0.032	−0.313	−0.183	<0.001
Conditional effect of social support on depressive symptoms					
Social support, −1 SD	4.107	0.110	3.891	4.322	<0.001
Social support, average	3.434	0.090	3.258	3.611	<0.001
Social support, +1 SD	2.762	0.125	2.517	3.007	<0.001
Conditional effect of optimism on depressive symptoms					
Optimism, −1 SD	3.955	0.104	3.751	4.159	<0.001
Optimism, average	3.189	0.834	3.015	3.343	<0.001
Optimism, +1 SD	2.403	0.115	2.178	2.628	<0.001
Path 2: Negative life events → depressive symptoms					
Outcome depressive symptoms [R^2^ = 0.273; *F*(6;6,752) = 423.222]					
Negative life events	1.225	0.145	0.940	1.509	<0.001
Some yoga/meditation use	−0.130	0.191	−0.504	0.244	0.496
Frequent yoga/meditation use	−0.106	0.231	−0.559	0.348	0.647
Negative life events × some yoga/meditation use	−0.133	0.328	−0.776	0.510	0.685
Negative life events × frequent yoga/meditation use	−0.924	0.382	−1.672	−0.176	0.016
Conditional effect of yoga on depressive symptoms					
No yoga/meditation use	1.225	0.145	0.940	1.509	<0.001
Some yoga/meditation use	1.092	0.296	0.512	1.672	<0.001
Frequent yoga/meditation use	0.301	0.355	−0.395	0.996	0.397

*N* = 6,759 (women were excluded due to missing data); B = Regression coefficient, SE = standard error, BC=Bias corrected.

### 3.2. Mediating effects of stress

Table 3 (path 1) and Figure 1 show that the association between the presence of at least one negative life event and higher levels of depressive symptoms was confirmed via linear regression (unstandardized beta coefficient [β] = 2.21, 95% confidence interval [CI] 1.94 to 2.49, *p* < 0.001). Further analyses showed that the association between negative life events and perceived stress was statistically significant (β = 0.27; 95%CI 0.23 to 0.30, *p* < 0.001), as was the association between stress and depressive symptoms (β = 4.42; 95%CI 4.10 to 4.42, *p* < 0.001). The association between negative life events and depressive symptoms remained significant but was diminished when perceived stress was also included in the predictive model (β = 1.13; 95%CI 0.98 to 1.28). These results suggest that the relationship between negative life events and depression was only partially mediated through perceived stress. The mediation through stress explained more than 50% of the total effect.

### 3.3. Moderating effects of social support, optimism

Further analyses showed that social support and optimism independently predicted stress (Social support: β = −0.14; 95%CI −0.16 to −0.12; optimism: β = −0.05; 95%CI −0.06 to −0.05), but there was no moderation on the pathway between negative life events and perceived stress. Both variables significantly moderated the effect of perceived stress on depression (Social support x perceived stress: β = −0.38; 95%CI −0.54 to −0.23; optimism x perceived stress: β = −0.25; 95%CI −0.31 to −0.19, *p* < 0.001).

### 3.4. Moderating effects of yoga/meditation use

Table 3 (path 2) and Figure 1 show that yoga/meditation use did not moderate the effect of perceived stress on depression; instead, the use of yoga/ meditation was found to moderate the direct effect between negative life events and depressive symptoms (β = −0.92; 95%CI, −1.67 to −0.18, *p* = 0.02). In women who were practicing yoga/meditation “often,” negative life events were not associated with depressive symptoms, whereas in women who did not practice yoga/meditation at all, depressive symptoms were higher when women experienced negative life events.

**Figure 2:**
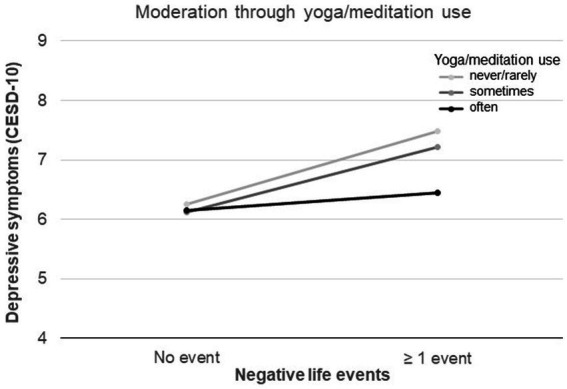
Moderating effects of yoga/meditation on the relationship between negative life events and depressive symptoms.

The moderating effect of yoga/meditation practice on the relationship between negative life events and depressive symptoms is further shown in Figure 2, indicating that the “often” use of yoga/meditation dampens the influence between life events and depressive symptoms intensity, compared to “never/rarely” use.

## 4. Discussion

This study presents several key findings. First of all, this study identified perceived stress as a mediator between the occurrence of negative life events, and depression symptoms. Secondly, the relationship between perceived stress and depressive symptoms is moderated by the amount of social support and optimism. Lastly, the use of yoga/meditation moderated the direct path between negative life events and depression, but not the indirect path through perceived stress. No moderating influence of physical activity was found.

Previous research has documented a relationship between negative life events and major depressive episodes (Kendler et al., 1999; Hammen, 2005; Risch et al., 2009) and has also indicated that the presence of negative life events *per se* may not be sufficient to induce depression, and that the response may be influenced by the perception of stress (Kuiper et al., 1986). High levels of perceived stress may not only lead to lowered mood, irritability, and disrupted sleep, but people under stress also may neglect healthy lifestyle practices which itself may contribute to depressive mood (Seib et al., 2014).

Our study also finds a moderating effect of social support on depression, “buffering” the impact of perceived stress on depressive symptoms. The impact of social support has been studied before extensively, and a number of studies confirms the role of positive social circumstances in the development of stress disorders and depression (Siegel and Brown, 1988; Auerbach et al., 2011; Marroquin, 2011). Studies have also indicated that the presence of social support can reduce the sympathetic response to stress (Taylor, 2011), however findings are inconsistent as some studies did not find beneficial effects of social support (Auerbach et al., 2011; Taylor, 2011), and mechanisms are not well understood (Marroquin, 2011). Furthermore an optimistic view on life was also found to moderate the relationship between stress and depression in our study. Optimism is highly correlated with an active, problem-focused coping style (M.F. Scheier and Carver, 1992), which in turn may serve to mitigate feelings of helplessness and thus reduce perceived stress. Research has also shown that optimism is strongly associated with resilience and the successful adaption to adverse circumstances (Souri and Hasanirad, 2011).

Given that physical activity may serve to prevent the onset of depression (Mammen and Faulkner, 2013), we were surprised that physical activity did not moderate the impact of stressful life events on depression. Previous research is mixed. For example, in an epidemiologic study of young adults in Canada, physical activity did buffer the effect of work stress, but not personal stress, financial stress, or recent life events, on subsequent depression in early adulthood (Colman et al., 2014). An epidemiologic study of women in the US found that physical activity did decrease the association between recent chronic stress and depressive symptoms, but only in African American (not white) women (Lincoln, 2017). Finally, other research in a large sample of women in the US either failed to find that physical activity served as a moderator of specific stressors and depression, or found that the effect was very small (Uebelacker et al., 2013).

Unlike physical activity, some yoga and meditation practices may be employed at any moment, and as part of an immediate response to stressful life events (Uebelacker et al., 2017). That is, one cannot always leave a situation and go for a walk or a run, but one may always direct one’s attention to taking long slow breaths, or one may silently repeat a mantra. Further, in many yoga classes, aspects of mindfulness are taught; where students are taught to step back and observe and accept (rather than immediately react) their own thoughts, feelings, or sensations. Gard et al. propose that yoga improves self-regulation, which would include the management of emotional, cognitive, and behavioral responses when one encounters stressful events in everyday life (Gard et al., 2014). These skills are very relevant in moments of stress, and may help to prevent the development of depression (van der Velden et al., 2015). Evidence from systematic reviews found some evidence that yoga and meditation might be beneficial for depression (Cramer et al., 2013a, 2017; Jain et al., 2015), although there is limited evidence for effects when yoga as an add-on to antidepressant medication was compared to medication alone, and the risk–benefit ratio of yoga for depression is unclear (Cramer et al., 2017).

The reduction of stress is one of the most popular and plausible modes of action (Chiesa and Serretti, 2009; Uebelacker et al., 2010). However our analysis found no influence of yoga/meditation practice on the perceived stress mediated pathway. Instead our study found that regular yoga/meditation use directly “dampened” the association between negative life events and depressive symptoms; where those women practicing yoga “often” had lower CESD-10 scores on average when reporting negative life events, compared to women without any yoga practice.

It is plausible that yoga not only regulates the cognitive aspects of stress (i.e., perceived stress), but may have a positive impact on other bodily functions such as sleep, and appetite, which might be disrupted as a consequence of experiencing negative life events (Lavie, 2001; Keller et al., 2007). It is also possible that yoga or meditation practice helps to divert individuals from other types of negative cognition (such as self-criticism) through helping the practitioner focus on posture and breathing during yoga classes and beyond (Ramel et al., 2004; Chiesa and Serretti, 2009; Kinser et al., 2013a,b). These hypotheses are speculative, and need to be examined in future research to uncover the full potential of yoga and meditation for depression prevention and management, and their effects need to be distinguished.

Because these data are cross-sectional, the findings do not necessarily establish a causal relationship with yoga practice improving resilience when life events occur. Other explanations are possible; such as women who are more resilient and experience negative life events are more likely to practice yoga regularly as part of their active coping style. At this stage, more research is warranted to identify the ways in which yoga/meditation may be linked to resilience when negative life events occur.

Other psychological variables that have been discussed as mechanisms for the effects of yoga and meditation are self-efficacy expectations, body awareness, mindfulness, and spirituality (Cramer et al., 2013b; Gard et al., 2014; Shonin and Van Gordon, 2016; Park et al., 2020). Future studies should investigate a potential mediator or moderator function of these variables.

### 4.1. Strengths and limitations

The ALSWH is a comprehensive and well-respected source for epidemiological data with a large number of participants. There are however some limitations: due to the structure of the survey, which was not specifically designed to address this specific research question, yoga/meditation practice was investigated as a single item; therefore no conclusive judgement regarding yoga or meditation practice as separate practices can be made. This is a clear limitation, since meditation can be part of a yoga practice and yoga is sometimes subsumed under meditation, but not every yoga practice has to include meditation and clearly not every form of meditation can be categorized as part of the yoga tradition. Future studies should also assess the practiced yoga style, the reason why (fitness, trend, or spiritual search, focus on mindfulness) and how (frequency, duration, style, use of meditation and/or breathing techniques or not, individual or group setting) it is practiced. The same is true for meditation.

Similarly, the questions on negative life events were vague and did not include, for example, an assessment of the frequency of such events. However, the latter could have a major impact on the effects of negative life events on mental health. Data are based on self-reports, and women may not have recollected all details correctly. A social desirability bias cannot be ruled out. The dichotomous variable for life events did not distinguish between the nature of various types (or numbers) of life events, however, the simplification was necessary to have an adequate number for statistical analyses. Similarly, depression was measured only as current depression by questionnaire. Although different relationships are expected for acute vs. chronic depression, this could not be differentiated due to lack of data. Finally, a number of other unmeasured variables may have influenced the relationship between life events and depression. Future research should not only use longitudinal designs, but collect data on yoga/meditation use, and its association with negative life events, stress and depression in much more depth.

## 5. Conclusion

This analysis assessed the mechanism of yoga and meditation for depression prevention. Yoga/meditation use was a significant moderator of the relationship between negative life events and depression. Rather than moderating its effects through stress, yoga/meditation seems to modify a more direct path on depression. Further studies are warranted to further evaluate the mechanisms of yoga/meditation for depression prevention.

## Data availability statement

The data analyzed in this study is subject to the following licenses/restrictions: ALSWH survey data are owned by the Australian Government Department of Health and due to the personal nature of the data collected, release by ALSWH is subject to strict contractual and ethical restrictions. Ethical review of ALSWH is by the Human Research Ethics Committees at The University of Queensland and The University of Newcastle. De-identified data are available to collaborating researchers where a formal request to make use of the material has been approved by the ALSWH Data Access Committee. The committee is receptive of requests for datasets required to replicate results. Information on applying for ALSWH data is available from https://alswh.org.au/for-data-users/applying-for-data/. Requests to access these datasets should be directed to alswh@uq.edu.au.

## Ethics statement

The studies involving humans were approved by University of Newcastle’s Human Research Ethics Committee, approval numbers: H-076-0795 and H-2012-0256, and the University of Queensland’s Medical Research Ethics Committee, approval numbers: 2004000224 and 2012000950. The studies were conducted in accordance with the local legislation and institutional requirements. The participants provided their written informed consent to participate in this study.

## Author contributions

RL, DA, and HC contributed to conception and design of the study. RL, DA, and DS performed the statistical analysis. RL wrote the first draft of the manuscript. DA, LU, DS, JA, and HC wrote sections of the manuscript. All authors contributed to the article and approved the submitted version.

## Funding

This work was supported by an Australian Research Council Discovery Project (DP140100238), and we are also grateful to the ARC for supporting Distinguished JA via a Professorial Future Fellowship (FT140100195) while working on this manuscript. We acknowledge support by Open Access Publishing Fund of University of Tübingen.

## Conflict of interest

The authors declare that the research was conducted in the absence of any commercial or financial relationships that could be construed as a potential conflict of interest.

## Publisher’s note

All claims expressed in this article are solely those of the authors and do not necessarily represent those of their affiliated organizations, or those of the publisher, the editors and the reviewers. Any product that may be evaluated in this article, or claim that may be made by its manufacturer, is not guaranteed or endorsed by the publisher.

## References

[ref1] Auerbach R. P. Bigda-Peyton J. S. Eberhart N. K. Webb C. A. Ho M. H. (2011). Conceptualizing the prospective relationship between social support, stress, and depressive symptoms among adolescents. J. Abnorm. Child Psychol. 39, 475–487. doi: 10.1007/s10802-010-9479-x, PMID: 21188628

[ref2] Baron R. M. Kenny D. A. (1986). The moderator-mediator variable distinction in social psychological research: conceptual, strategic, and statistical considerations. J. Pers. Soc. Psychol. 51, 1173–1182. doi: 10.1037/0022-3514.51.6.1173 3806354

[ref3] Bell S. Lee C. (2003). Perceived stress revisited: the Women’s health Australia project young cohort. Psychol. Health Med. 8, 343–353. doi: 10.1080/1354850031000135786

[ref4] Brown W. J. Bauman A. Bull F. Burton N. W. (2013). “Development of evidence-based physical activity recommendations for adults (18-64 years)” in Report prepared for the Australian Government Department of Health (Canberra, Australia: Australian Department of Health)

[ref5] Brown W. J. Bryson L. Byles J. E. Dobson A. J. Lee C. Mishra G.. (1998). Women’s health Australia: recruitment for a national longitudinal cohort study. Women Health 28, 23–40. doi: 10.1300/J013v28n01_03, PMID: 10022055

[ref6] Brown W. J. Burton N. W. Marshall A. L. Miller Y. D. (2008). Reliability and validity of a modified self-administered version of the active Australia physical activity survey in a sample of mid-age women. Aust. N. Z. J. Public Health 32, 535–541. doi: 10.1111/j.1753-6405.2008.00305.x, PMID: 19076744

[ref7] Brown W. J. Dobson A. J. Bryson L. Byles J. E. (1999). Women’s health Australia: on the progress of the main cohort studies. J. Womens Health Gend. Based Med. 8, 681–688. doi: 10.1089/jwh.1.1999.8.681 10839654

[ref8] Chiesa A. Serretti A. (2009). Mindfulness-based stress reduction for stress management in healthy people: a review and meta-analysis. J. Altern. Complement. Med. 15, 593–600. doi: 10.1089/acm.2008.0495, PMID: 19432513

[ref9] Colman I. Zeng Y. McMartin S. E. Naicker K. Ataullahjan A. Weeks M.. (2014). Protective factors against depression during the transition from adolescence to adulthood: findings from a national Canadian cohort. Prev. Med. 65, 28–32. doi: 10.1016/j.ypmed.2014.04.008, PMID: 24732721

[ref10] Cramer H. (2015). Yoga in Germany - results of a nationally representative survey. Forsch. Komplementmed. 22, 304–310. doi: 10.1159/000439468, PMID: 26565982

[ref11] Cramer H. Anheyer D. Lauche R. Dobos G. (2017). A systematic review of yoga for major depressive disorder. J. Affect. Disord. 213, 70–77. doi: 10.1016/j.jad.2017.02.006, PMID: 28192737

[ref12] Cramer H. Anheyer D. Saha F. J. Dobos G. (2018). Yoga for posttraumatic stress disorder - a systematic review and meta-analysis. BMC Psychiatry 18:72. doi: 10.1186/s12888-018-1650-x, PMID: 29566652 PMC5863799

[ref13] Cramer H. Hall H. Leach M. Frawley J. Zhang Y. Leung B.. (2016a). Prevalence, patterns, and predictors of meditation use among US adults: a nationally representative survey. Sci. Rep. 6:36760. doi: 10.1038/srep36760, PMID: 27829670 PMC5103185

[ref14] Cramer H. Lauche R. Haller H. Langhorst J. Dobos G. Berger B. (2013a). “I’m more in balance”: a qualitative study of yoga for patients with chronic neck pain. J. Altern. Complement. Med. 19, 536–542. doi: 10.1089/acm.2011.0885, PMID: 23336342

[ref15] Cramer H. Lauche R. Langhorst J. Dobos G. (2013b). Yoga for depression: a systematic review and meta-analysis. Depress. Anxiety 30, 1068–1083. doi: 10.1002/da.22166 23922209

[ref16] Cramer H. Ward L. Steel A. Lauche R. Dobos G. Zhang Y. (2016b). Prevalence, patterns, and predictors of yoga use: results of a U.S. nationally representative survey. Am. J. Prev. Med. 50, 230–235. doi: 10.1016/j.amepre.2015.07.037, PMID: 26497261

[ref17] De Michelis E. (2008). “Modern yoga: history and forms” in Yoga in the modern world. eds. Singleton M. Byrne J. (London; New York: Routledge), 29–47.

[ref18] Feuerstein G. (1998). The yoga tradition: Prescott: Hohm Press.

[ref19] Gard T. Noggle J. J. Park C. L. Vago D. R. Wilson A. (2014). Potential self-regulatory mechanisms of yoga for psychological health. Front. Hum. Neurosci. 8:770. doi: 10.3389/fnhum.2014.00770, PMID: 25368562 PMC4179745

[ref20] Hammen C. (2005). Stress and depression. Annu. Rev. Clin. Psychol. 1, 293–319. doi: 10.1146/annurev.clinpsy.1.102803.143938 17716090

[ref21] Hayes A. F. (2009). Beyond Baron and Kenny: statistical mediation analysis in the new millennium. Commun. Monogr. 76, 408–420. doi: 10.1080/03637750903310360

[ref22] Hayes A. F. (2017). Introduction to mediation, moderation, and conditional process analysis: A regression-based approach. New York; London: Guilford Publications.

[ref23] Heissel A. Heinen D. Brokmeier L. L. Skarabis N. Kangas M. Vancampfort D.. (2023). Exercise as medicine for depressive symptoms? A systematic review and meta-analysis with meta-regression. Br. J. Sports. Med 57, 1049–1057. doi: 10.1136/bjsports-2022-106282, PMID: 36731907 PMC10423472

[ref24] Holden L. Lee C. Hockey R. Ware R. S. Dobson A. J. (2014). Validation of the MOS social support survey 6-item (MOS-SSS-6) measure with two large population-based samples of Australian women. Qual. Life Res. 23, 2849–2853. doi: 10.1007/s11136-014-0741-5, PMID: 24962651

[ref25] Irandoust K. Taheri M. Chtourou H. Nikolaidis P. T. Rosemann T. Knechtle B. (2019). Effect of time-of-day-exercise in group settings on level of mood and depression of former elite male athletes. Int. J. Environ. Res. Public Health 16:3541. doi: 10.3390/ijerph16193541, PMID: 31546685 PMC6801561

[ref26] Jain F. A. Walsh R. N. Eisendrath S. J. Christensen S. Rael Cahn B. (2015). Critical analysis of the efficacy of meditation therapies for acute and subacute phase treatment of depressive disorders: a systematic review. Psychosomatics 56, 140–152. doi: 10.1016/j.psym.2014.10.007, PMID: 25591492 PMC4383597

[ref27] Jia Y. Wang X. Cheng Y. (2020). Relaxation therapy for depression: an updated Meta-analysis. J. Nerv. Ment. Dis. 208, 319–328. doi: 10.1097/NMD.0000000000001121 32221187

[ref28] Keller M. C. Neale M. C. Kendler K. S. (2007). Association of different adverse life events with distinct patterns of depressive symptoms. Am. J. Psychiatry 164, 1521–1529. doi: 10.1176/appi.ajp.2007.06091564, PMID: 17898343

[ref29] Kendler K. S. Karkowski L. M. Prescott C. A. (1999). Causal relationship between stressful life events and the onset of major depression. Am. J. Psychiatry 156, 837–841. doi: 10.1176/ajp.156.6.837 10360120

[ref30] Kinser P. A. Bourguignon C. Taylor A. G. Steeves R. (2013a). “A feeling of connectedness”: perspectives on a gentle yoga intervention for women with major depression. Issues Ment. Health Nurs. 34, 402–411. doi: 10.3109/01612840.2012.762959, PMID: 23805925 PMC3703865

[ref31] Kinser P. A. Bourguignon C. Whaley D. Hauenstein E. Taylor A. G. (2013b). Feasibility, acceptability, and effects of gentle Hatha yoga for women with major depression: findings from a randomized controlled mixed-methods study. Arch. Psychiatr. Nurs. 27, 137–147. doi: 10.1016/j.apnu.2013.01.003, PMID: 23706890 PMC3664951

[ref32] Kuiper N. A. Olinger L. J. Lyons L. M. (1986). Global perceived stress level as a moderator of the relationship between negative life events and depression. J. Hum. Stress. 12, 149–153. doi: 10.1080/0097840x.1986.9936781, PMID: 3559198

[ref33] Lavie P. (2001). Sleep disturbances in the wake of traumatic events. N. Engl. J. Med. 345, 1825–1832. doi: 10.1056/NEJMra012893 11752360

[ref34] Lincoln K. D. (2017). Social stress, obesity, and depression among women: clarifying the role of physical activity. Ethn. Health 24, 662–678. doi: 10.1080/13557858.2017.1346190, PMID: 28669235

[ref35] Mammen G. Faulkner G. (2013). Physical activity and the prevention of depression: a systematic review of prospective studies. Am. J. Prev. Med. 45, 649–657. doi: 10.1016/j.amepre.2013.08.001 24139780

[ref36] Marroquin B. (2011). Interpersonal emotion regulation as a mechanism of social support in depression. Clin. Psychol. Rev. 31, 1276–1290. doi: 10.1016/j.cpr.2011.09.005, PMID: 21983267

[ref37] Park C. L. Finkelstein-Fox L. Groessl E. J. Elwy A. R. Lee S. Y. (2020). Exploring how different types of yoga change psychological resources and emotional well-being across a single session. Complement. Ther. Med. 49:102354. doi: 10.1016/j.ctim.2020.102354, PMID: 32147083 PMC7081324

[ref38] Preacher K. J. Hayes A. F. (2004). SPSS and SAS procedures for estimating indirect effects in simple mediation models. Behav. Res. Methods Instrum. Comput. 36, 717–731. doi: 10.3758/BF03206553, PMID: 15641418

[ref39] Preacher K. J. Hayes A. F. (2008). Asymptotic and resampling strategies for assessing and comparing indirect effects in multiple mediator models. Behav. Res. Methods 40, 879–891. doi: 10.3758/BRM.40.3.879, PMID: 18697684

[ref40] Radloff L. S. (1977). The CES-D scale: a self-report depression scale for research in the general population. Appl. Psychol. Measurment. 1, 385–401. doi: 10.1177/014662167700100306

[ref41] Ramel W. Goldin P. R. Carmona P. E. McQuaid J. R. (2004). The effects of mindfulness meditation on cognitive processes and affect in patients with past depression. Cognitive Ther. Res. 28, 433–455. doi: 10.1023/B:COTR.0000045557.15923.96

[ref42] Risch N. Herrell R. Lehner T. Liang K. Y. Eaves L. Hoh J.. (2009). Interaction between the serotonin transporter gene (5-HTTLPR), stressful life events, and risk of depression: a meta-analysis. JAMA 301, 2462–2471. doi: 10.1001/jama.2009.878, PMID: 19531786 PMC2938776

[ref43] Scheier M. F. Carver C. S. (1985). Optimism, coping, and health: assessment and implications of generalized outcome expectancies. Health Psychol. 4, 219–247. doi: 10.1037/0278-6133.4.3.219, PMID: 4029106

[ref44] Scheier M. F. Carver C. S. (1992). Effects of optimism on psychological and physical well-being: theoretical overview and empirical update. Cognit. Ther. Res. 16, 201–228. doi: 10.1007/BF01173489

[ref45] Scheier M. F. Carver C. S. Bridges M. W. (1994). Distinguishing optimism from neuroticism (and trait anxiety, self-mastery, and self-esteem): a reevaluation of the life orientation test. J. Pers. Soc. Psychol. 67, 1063–1078. doi: 10.1037/0022-3514.67.6.1063, PMID: 7815302

[ref46] Seib C. Whiteside E. Lee K. Humphreys J. Tran T. H. Chopin L.. (2014). Stress, lifestyle, and quality of life in midlife and older Australian women: results from the stress and the health of women study. Womens Health Issues 24, e43–e52. doi: 10.1016/j.whi.2013.11.004, PMID: 24439946

[ref47] Shonin E. Van Gordon W. (2016). The mechanisms of mindfulness in the treatment of mental illness and addiction. Int. J. Ment. Health. Addict 14, 844–849. doi: 10.1007/s11469-016-9653-7, PMID: 27688740 PMC5023750

[ref48] Siegel J. M. Brown J. D. (1988). A prospective study of stressful circumstances, illness symptoms, and depressed mood among adolescents. Dev. Psychol. 24, 715–721. doi: 10.1037/0012-1649.24.5.715

[ref49] Souri H. Hasanirad T. (2011). Relationship between resilience, optimism and psychological well-being in students of medicine. Procedia. Soc. Behav. Sci. 30, 1541–1544. doi: 10.1016/j.sbspro.2011.10.299

[ref50] Taylor S. E. (2011). Social support: a review. The Handbook of Health Psychology. 189:214. doi: 10.1093/oxfordhb/9780195342819.013.0009

[ref51] Uebelacker L. A. Eaton C. B. Weisberg R. Sands M. Williams C. Calhoun D.. (2013). Social support and physical activity as moderators of life stress in predicting baseline depression and change in depression over time in the Women’s Health Initiative. Soc. Psychiatry Psychiatr. Epidemiol. 48, 1971–1982. doi: 10.1007/s00127-013-0693-z, PMID: 23644722 PMC3796164

[ref52] Uebelacker L. A. Epstein-Lubow G. Gaudiano B. A. Tremont G. Battle C. L. Miller I. W. (2010). Hatha yoga for depression: critical review of the evidence for efficacy, plausible mechanisms of action, and directions for future research. J. Psychiatr. Pract. 16, 22–33. doi: 10.1097/01.pra.0000367775.88388.96, PMID: 20098228

[ref53] Uebelacker L. A. Kraines M. Broughton M. K. Tremont G. Gillette L. T. Epstein-Lubow G.. (2017). Perceptions of hatha yoga amongst persistently depressed individuals enrolled in a trial of yoga for depression. Complement. Ther. Med. 34, 149–155. doi: 10.1016/j.ctim.2017.06.008, PMID: 28917367 PMC5679431

[ref54] van der Velden A. M. Kuyken W. Wattar U. Crane C. Pallesen K. J. Dahlgaard J.. (2015). A systematic review of mechanisms of change in mindfulness-based cognitive therapy in the treatment of recurrent major depressive disorder. Clin. Psychol. Rev. 37, 26–39. doi: 10.1016/j.cpr.2015.02.001, PMID: 25748559

[ref55] Xue C. C. Zhang A. L. Lin V. Da Costa C. Story D. F. (2007). Complementary and alternative medicine use in Australia: a national population-based survey. J. Altern. Complement. Med. 13, 643–650. doi: 10.1089/acm.2006.6355, PMID: 17718647

